# Flow-induced bending of flagella restricts *Pseudomonas aeruginosa* surface departure

**DOI:** 10.1128/mbio.02740-25

**Published:** 2025-12-12

**Authors:** Jessica-Jae S. Palalay, Joseph E. Sanfilippo

**Affiliations:** 1 Department of Biochemistry, University of Illinois at Urbana-Champaign 14589, Urbana, Illinois, USA; Dartmouth College, Hanover, New Hampshire, USA

**Keywords:** flow, shear force, flagella, *Pseudomonas aeruginosa*, microfluidics

## Abstract

Shear force associated with flow can bend, twist, or stretch cellular appendages. Bacterial appendages such as flagella can rotate and generate forces. However, it is unclear how environmental and cell-generated forces interact as bacteria associate with surfaces. Here, we use microfluidics, flagellar labeling, and genetics to discover that flow bends flagella to restrict surface departure of the human pathogen *Pseudomonas aeruginosa*. By imaging wild-type cells with blocked flagella and using mutants with paralyzed flagella, we demonstrate that flagellar rotation force promotes surface departure in host-relevant shear regimes. Our single-cell experiments reveal two distinct subpopulations in flow: cells with their flagellum positioned upstream and cells with their flagellum positioned downstream. By independently modulating flow and solution viscosity, we show that shear force bends upstream flagella around the cell and blocks rotation within seconds after surface arrival. In contrast, downstream flagella can continue to rotate after surface arrival. Cells with downstream flagella depart the surface more frequently than cells with upstream flagella, indicating how flow direction can determine bacterial cell fate on surfaces. Together, our results demonstrate how the geometric relationship between flow and cell appendages can generate subpopulations and control surface behaviors.

**IMPORTANCE:**

Bacteria use cell appendages such as flagella to interact with their environment. While bacteria are typically studied in conditions that lack flow, host environments are often flowing. In this study, we use microfluidic devices to test how host-relevant flow impacts flagella of the human pathogen *Pseudomonas aeruginosa*. As each *P. aeruginosa* cell has only one flagellum, we observed cells with their flagellum facing upstream and cells with their flagellum facing downstream. We discover that while shear force bends upstream flagella and prevents their rotation, downstream flagella can continue to rotate. As a consequence, cells with downstream flagella are more likely to depart the surface. Our results reveal a mechanism by which shear force can impact the surface behavior of a bacterial pathogen.

## INTRODUCTION

Mechanical features of the environment impact bacterial behavior (1–3). For example, pathogenic bacteria are influenced by shear forces and surface association when infecting heart valves or catheters (4–6). Recent discoveries have led to the emerging paradigm that bacteria can respond to environmental mechanics (2, 7–10). As the cell wall makes bacterial cells rigid, researchers hypothesized that bacterial mechanosensing is mediated by flexible appendages such as flagella and type IV pili (7, 11–16). While there is growing evidence that flagella and type IV pili control mechanical responses (17–20), it remains unclear how mechanical forces directly impact bacterial appendages. Considering that mechanics play an important role in shaping host-pathogen interactions, there is a critical need to understand how mechanical forces impact bacterial behavior.

Conceptually, there are at least three ways that environmental mechanics could impact bacterial behavior (2, 3). First, a mechanical stimulus could be sensed by a cell surface component, which could lead to changes in internal signaling. There is evidence that flagella and type IV pili use this mechanism to sense surface contact and trigger surface-associated behaviors (14, 16–18). Second, a mechanical stimulus could impact the local concentration of small molecules, which could be sensed by the cell and trigger internal signaling. There is evidence that shear rate impacts behavior by replenishing or washing away small molecules such as autoinducers or hydrogen peroxide, which ultimately are sensed by the cell (9, 21–23). Third, a mechanical stimulus could induce a conformational change in the cell or a cell surface component that subsequently impacts behavior without altering internal signaling. There is evidence that shear force modulates adhesion by tipping cells over or stretching a cell surface adhesin that displays catch bond properties (24–26). As there is evidence for many forms of bacterial mechanoregulation, it is important to consider various possibilities when characterizing a bacterial response.

During infection, bacteria must contend with host-generated fluid flow (4, 5, 27). Using microfluidic systems, recent studies demonstrated how flow impacts antimicrobial resistance (28), quorum sensing (22, 29), gene expression (9, 14, 21, 23), and surface motility (25, 26, 30). Perhaps, the most obvious impact of flow is on adhesion, where the shear force associated with flow can remove cells from a surface. However, the study of adhesion in flow revealed the counterintuitive result that increasing flow often enhances adhesion (25, 26, 31, 32). In lower shear regimes, *Pseudomonas aeruginosa* cells use type IV pilus retraction to promote surface departure by tilting themselves away from the surface (26). Higher shear forces overcome pilus-dependent tilting, push *P. aeruginosa* cells closer to the surface, and increase their surface residence time (26). This example highlights how the interaction between cell-generated forces and environmental forces can lead to unexpected outcomes.

To study the interaction between cell-generated forces and environmental forces, we examined how *P. aeruginosa* adhesion is impacted by flagellar rotation and flow. Using mutants with paralyzed flagella, we show that flagellar rotation promotes both surface arrival and departure in host-relevant shear regimes. Combining microfluidics and flagellar labeling, we demonstrate that flow can bend the flagellum and block rotation. By tracking single cells in flow, we discover two distinct subpopulations: cells with their flagellum positioned upstream and cells with their flagellum positioned downstream. By independently modulating flow intensity and solution viscosity, we establish that shear force bends upstream flagella and halts flagellar rotation. In contrast, downstream flagella are not bent by shear force and can continue to rotate after surface arrival. Importantly, cells with downstream flagella leave the surface more often than cells with upstream flagella. Collectively, our results reveal how the direction of environmental shear force interacts with the force of flagellar rotation to control bacterial surface behavior.

## RESULTS

**Fig 1:**
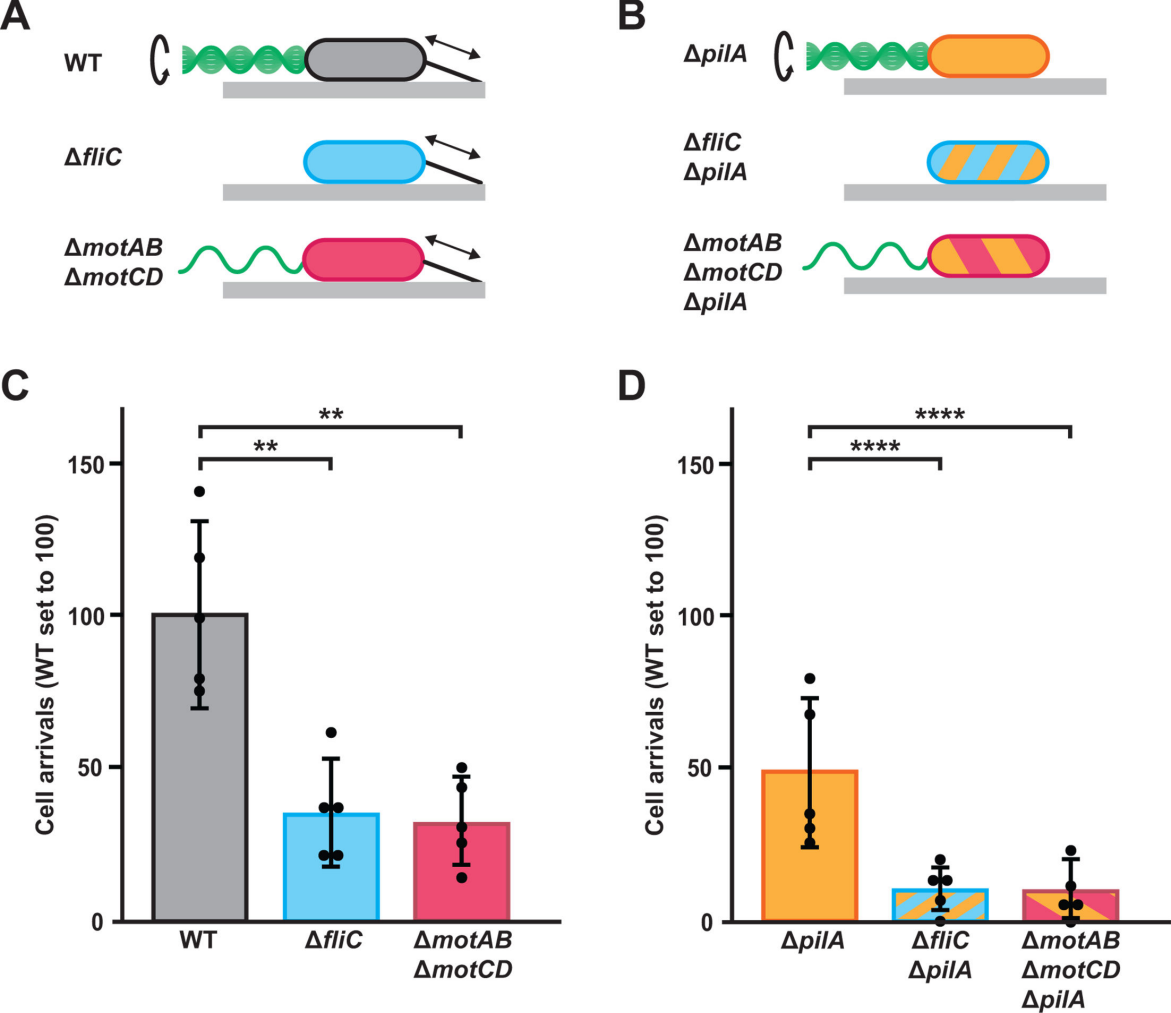
Flagellar rotation promotes *P. aeruginosa* surface arrival in flow. (**A, B**) Representation of wild-type (WT) and mutant strains used in this study. Cells either have a rotating flagellum (top row), lack a flagellum (middle row), or have a non-rotating flagellum (bottom row). Flagella in the diagram are located on the left side of the cells and are in green. Cells also have dynamic type IV pili (**A**) or lack type IV pili. (**B**) Pili in the diagram are located on the right side of the cells and are in black. (**C, D**) Quantification of cell arrivals for WT and mutant strains flowed into microfluidic devices at a shear rate of 800 s^−1^. Cell arrivals were determined by how many cells landed on the surface and remained attached for at least 2 s (to confirm the cell is attached to the surface). Measurements were taken over 30 s, and WT cell arrivals were normalized to 100. Statistical significance determined via five biological replicates using one-way ANOVA followed by Dunnett’s test: **, *P* < 0.01; ****, *P* < 0.0001.

To investigate how the flagellum impacts initial *P. aeruginosa* surface interactions, we generated mutant strains lacking functional flagella (Fig. 1A and B). To confirm our strains, we measured swimming motility with a traditional swim plate assay (Fig. S1). While wild-type (WT) cells were motile, Δ*fliC* mutant cells lacking the flagellar filament exhibited no motility (Fig. S1). Additionally, mutants lacking either flagellar stator complex (MotAB or MotCD) had decreased motility (Fig. S1), confirming that both stator complexes contribute to flagellar rotation (33–36). Consistent with previous reports (33–36), our mutant lacking both *motAB* and *motCD* exhibited no swimming motility (Fig. S1). Thus, we chose to use Δ*fliC* to examine the role of the flagellum and Δ*motAB* Δ*motCD* to examine the role of flagellar rotation.

To determine if flagellar rotation impacts surface arrival in flow, we loaded cells into a syringe at a mid-log concentration and introduced them into empty microfluidic channels at a shear rate of 800 s^−1^. We chose a shear rate of 800 s^−1^ as it falls in the range of host-relevant shear rates found in the urinary tract (27), lungs (4), and bloodstream (5). Surface arrival was recorded when a cell adhered to the surface and remained for at least 2 s (Fig. 1C and D; Fig. S2), ensuring that cells were surface-attached. Compared to WT, Δ*fliC* cells had approximately 65% less arrivals and Δ*motAB* Δ*motCD* cells had approximately 70% less arrivals (Fig. 1C). Thus, flagellar rotation (and not simply the presence of a flagellum) has a major role in *P. aeruginosa* surface arrival in flow. Type IV pili also have an important role in surface arrival (26). We confirmed this result using a Δ*pilA* mutant (which lacks the pilus filament) (Fig. 1B) and generated combinations of mutations to test the interaction between type IV pili and flagellar rotation (Fig. 1B). Compared to Δ*pilA* cells, Δ*fliC* Δ*pilA* had approximately 80% less arrivals and Δ*motAB* Δ*motCD* Δ*pilA* cells had approximately 80% less arrivals (Fig. 1D). Together, these results establish that flagellar rotation acts independently of type IV pili to promote *P. aeruginosa* surface arrival in flow.

**Fig 2:**
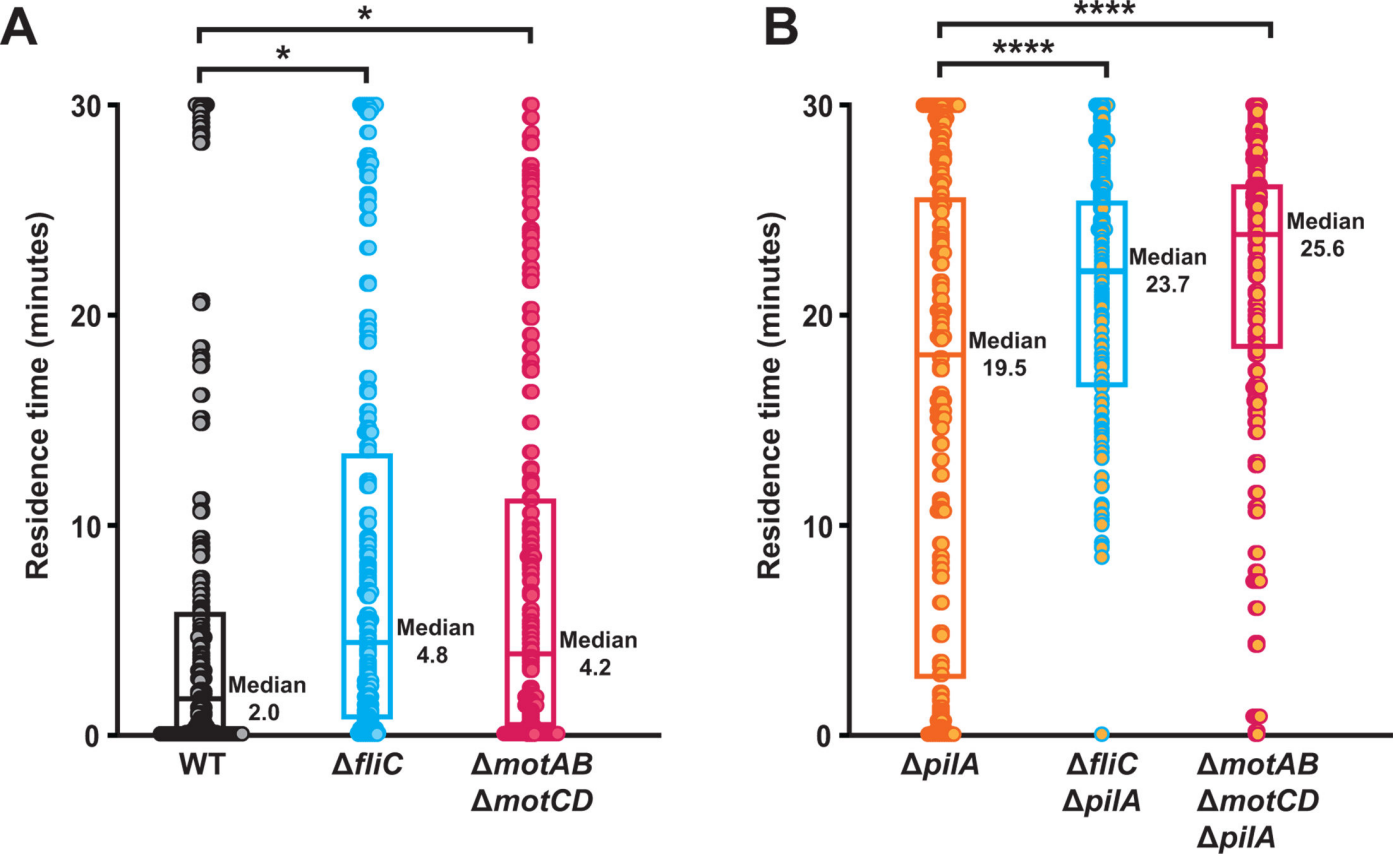
Flagellar rotation promotes *P. aeruginosa* surface departure in flow. Surface residence time of WT, flagellar mutant cells (**A**), and cells lacking type IV pili (**B**) flowed into microfluidic devices at a shear rate of 800 s^−1^. Residence time is the time between surface arrival and departure. Each data point represents the residence time of one cell. Quantification from three biological replicates and 150 cells (50 from each replicate) of each bacterial strain were chosen at random. Statistical significance was determined using one-way ANOVA followed by Dunnett’s test: *, *P* < 0.05; ****, *P* < 0.0001. Boxplot represents the 25th percentile, median, and 75th percentile for each strain.

Does flagellar rotation impact surface departure in flow? As a rotating flagellum generates mechanical force, we hypothesized that flagellar rotation has a role in surface departure. To quantify surface departure, we measured the surface residence time (the time

between surface arrival and departure) of cells exposed to a shear rate of 800 s^−1^. While WT cells had a short residence time (median = 2 min), Δ*fliC* cells (median = 4.8 min) and Δ*motAB* Δ*motCD* cells (median = 4.2 min) exhibited statistically longer residence times (Fig. 2A). Based on these results, we hypothesize that flagellar rotation has an important role in *P. aeruginosa* surface departure in flowing environments.

Our previous work established that type IV pili have an important role in surface departure (26). Confirming the role of type IV pili, Δ*pilA* cells (median = 19.5 min) had a much longer residence time than WT (Fig. 2B). Using our combination mutants, we tested how type IV pili and flagellar rotation interact during surface departure. Compared to Δ*pilA*, Δ*fliC* Δ*pilA* cells (median = 23.7 min) and Δ*motAB* Δ*motCD* Δ*pilA* cells (median = 25.6 min) exhibited statistically longer residence times (Fig. 2B). Thus, we conclude that type IV pili and flagellar rotation contribute independently to surface departure. When focusing on the first 2 min, we noticed that while 24% of Δ*pilA* cells departed, only 1% of Δ*fliC* Δ*pilA* cells and 2% of Δ*motAB* Δ*motCD* Δ*pilA* cells departed (Fig. S3). These experiments support the hypothesis that flagellar rotation promotes cell departure during early surface interactions in flow.

**Fig 3:**
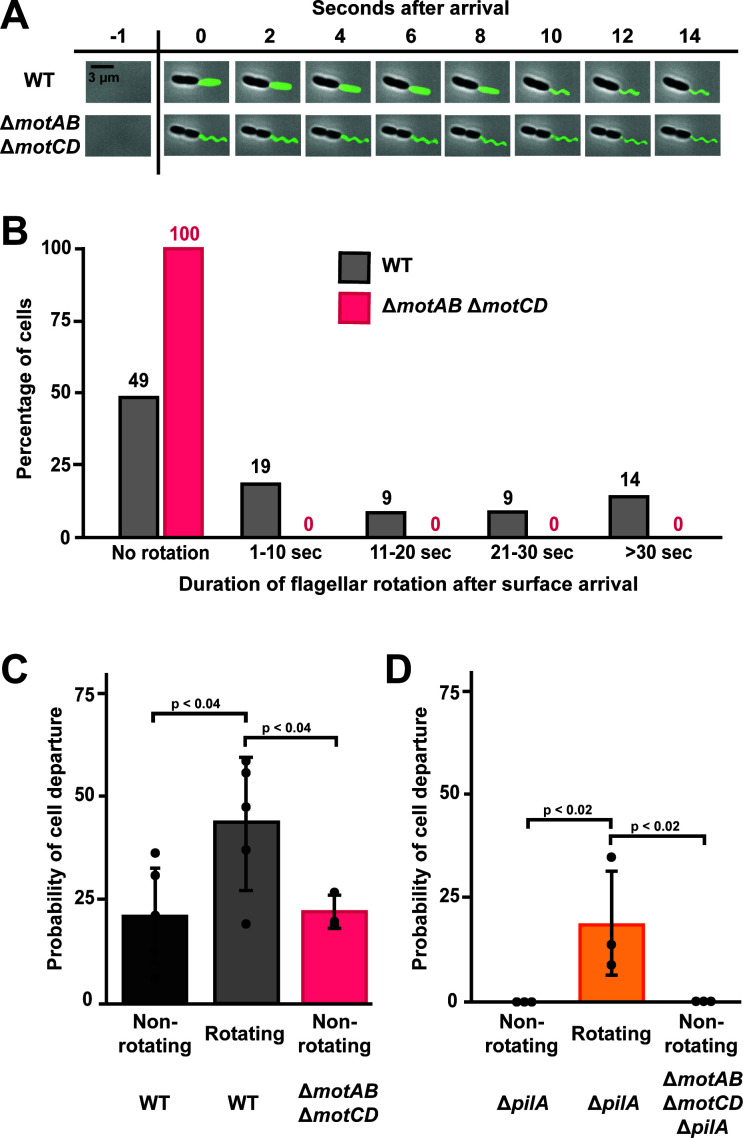
Cells with rotating flagella are more likely to depart the surface. (**A**) Representative images of surface-attached WT (top) and ∆*motAB* ∆*motCD* (bottom) cells with fluorescently labeled flagella. WT cell has a rotating flagellum, while ∆*motAB* ∆*motCD* cell does not have a rotating flagellum. Exposure time, 100 ms. Scale bar, 3 µm. (**B**) Duration of flagellar rotation after surface arrival of WT and ∆*motAB* ∆*motCD* cells. Five biological replicates were performed and a total of 550 WT cells and 125 ∆*motAB* ∆*motCD* cells were chosen for quantification and representation. Mean flagellar rotation of WT and ∆*motAB* ∆*motCD* cells are statistically different with *P* < 0.001. (**C**) Probability of surface departure of WT and ∆*motAB* ∆*motCD* cells. (**D**) Probability of surface departure of ∆*pilA* (orange line and shading) and ∆*motAB* ∆*motCD* ∆*pilA* (red line and orange & red shading) cells. All experiments were performed at a shear rate of 800 s^−1^. Cells were categorized as having rotating flagellum or non-rotating flagellum, and the percentage of cell departures was calculated. Quantification shows the average and standard deviations of at least three biological replicates. *P*-values were calculated with the student’s *t*-test.

Do surface-attached cells rotate their flagella? To visualize flagellar rotation of recently adhered *P. aeruginosa* cells, we introduced a cysteine point mutation (T394C) in the flagellar filament protein FliC (37, 38). Then, we used a thiol-reactive Alexa488 maleimide dye to fluorescently label the flagellum (37). We observed that the majority of cells (~70%) had 1 flagellum (Fig. S4) with a median length of 3.4 µm (Fig. S5). To examine flagellar rotation immediately after surface arrival, we introduced cells into a microfluidic device at a shear rate of 800 s^−1^ while simultaneously imaging fluorescently labeled flagella. As flagellar rotation is much faster (39–41) than the exposure time we used, we reasoned that rotating flagella would appear as rectangular-shaped blurs (Fig. 3A; Fig. S6). In contrast, non-rotating flagella appear in two dimensions as waveform shapes (Fig. 3A; Fig. S6). We observed that approximately 50% of WT flagella were not rotating 1 s after cell surface arrival (Fig. 3B). In contrast, the other 50% of WT flagella rotated for many seconds before stopping (Fig. 3B). Confirming the role of MotAB and MotCD in flagellar rotation, 100% of flagella from Δ*motAB* Δ*motCD* cells did not rotate (Fig. 3B). Our results establish that there are two types of recently adhered *P. aeruginosa* cells: those with rotating flagella and those with non-rotating flagella.

Do cells with rotating flagella have a higher frequency of surface departure? To examine the relationship between flagellar rotation and surface departure, we quantified the probability of surface departure of WT cells with rotating flagella and non-rotating flagella. We observed that WT cells with rotating flagella had a 45% chance of surface departure, while WT cells with non-rotating flagella only had a 20% chance (Fig. 3C). Consistent with the hypothesis that flagellar rotation promotes departure, Δ*motAB* Δ*motCD* cells (which lack flagellar rotation) had only a 21% chance of surface departure (Fig. 3C). We reasoned that the residual surface departure in cells with non-rotating flagella was due to the effects of type IV pili. In support of our hypothesis, 0% of Δ*pilA* cells with non-rotating flagella departed the surface, while 19% of Δ*pilA* cells with rotating flagella departed the surface (Fig. 3D). Similarly, 0% of Δ*motAB* Δ*motCD* Δ*pilA* cells (which lack flagellar rotation and type IV pili) departed the surface (Fig. 3D). Together, our results demonstrate that flagellar rotation promotes cell surface departure in flowing environments.

**Fig 4:**
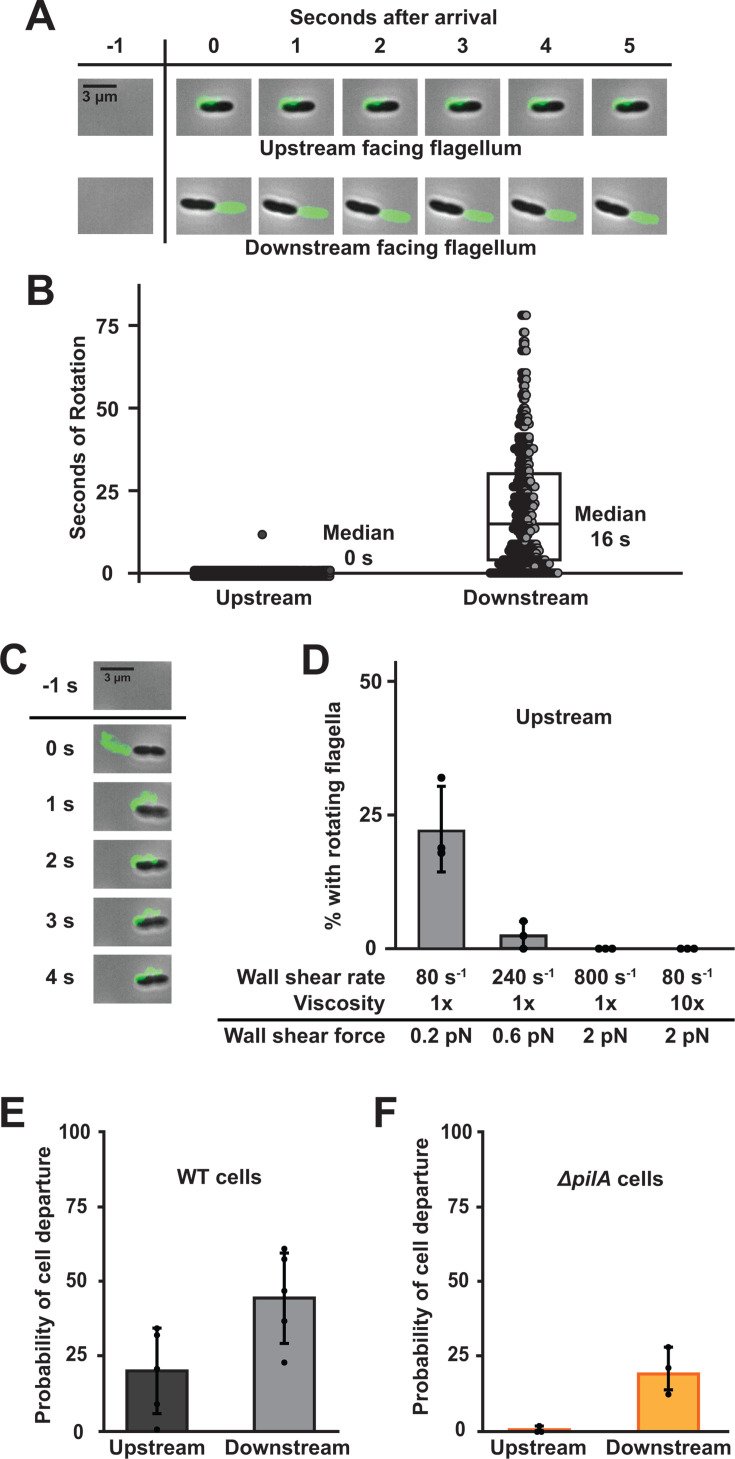
Shear force bends upstream facing flagella and blocks their rotation. (**A**) Representative images of surface-attached WT cells with an upstream facing flagellum or a downstream facing flagellum. Exposure time, 100 ms. Scale bar, 3 µm. (**B**) Duration of flagellar rotation of WT cells. Each data point represents one flagellum. Five biological replicates were performed, and 528 WT cells were chosen for quantification. Boxplot represents the 25th percentile, median, and 75th percentile. (**C**) Images of a cell as flow bends its flagellum. Scale bar, 3 µm. (**D**) Percentage of cells with upstream flagella that are rotating during exposure to different shear forces. Shear force was increased either by changing shear rate or fluid viscosity. Shear rate was modified by changing the flow rate of the syringe pump. 10× viscosity was generated by adding 15% Ficoll, which has been shown previously to modify local viscosity (9, 42). (**E**) Probability of cell departure of WT cells with upstream facing or downstream facing flagella. Mean cell departures were statistically different with *P* < 0.04. (**F**) Probability of cell departure of ∆*pilA* cells with upstream facing or downstream facing flagella. Mean cell departures were statistically different with *P* < 0.05. Quantification shows the average and SD of at least three biological replicates. Shear rate was 800 s^−1^, except where noted. *P*-values were calculated with the student’s *t*-test.

While imaging labeled flagella, we observed two distinct subpopulations of cells: cells with their flagella facing upstream and cells with their flagella facing downstream (Fig. 4A). We found that approximately 50% of WT cells had upstream flagella and 50% had downstream flagella (Fig. S7A). 73% of Δ*pilA* cells had upstream flagella and 27% had downstream flagella, suggesting type IV pili impact surface orientation during surface arrival (Fig. S7B). The 50/50 split in WT cells gave us a unique opportunity to examine how the directionality of flow impacts flagellar rotation. Flagella facing downstream rotated for a median of 16 s after cell surface arrival before stopping (Fig. 4B). In contrast, all flagella facing upstream were not rotating in the first frame after surface arrival (Fig. 4B). The sole exception was a cell that was shielded from flow by another tilted cell which was located directly in front of it (Fig. S8). Additionally, downstream facing flagella from ∆*motAB* and ∆*motCD* mutants exhibit rotation (Fig. S9), confirming either stator complex is sufficient for flagellar rotation. Together, these results lead us to the hypothesis that rotation of upstream facing flagella is rapidly blocked by flow, while downstream facing flagella can continue to rotate.

How does flow block rotation of upstream facing flagella? During our quantification of flagellar rotation time, we noticed that upstream flagella are bent around the cell body (Fig. 4A; Fig. S10). After closer examination, we observed that upstream flagella typically bend around the cell body in the first second after surface arrival (Fig. 4C). We hypothesized that flagellar bending was due to the shear force associated with flow. As shear force depends on shear rate and solution viscosity (9), we tested our hypothesis by independently modulating both variables. As increasing shear rate or viscosity increased the frequency of bent flagella, we concluded that flagellar bending was due to shear force (Fig. S10). To test if shear force is responsible for blocking rotation of upstream flagella, we quantified rotation while independently modulating shear rate and viscosity. While 23% of upstream flagella rotated at a shear force of 0.2 pN, only 3% of upstream flagella rotated when the shear force was increased to 0.6 pN (Fig. 4D). When the shear force was increased to 2 pN by increasing flow rate or by increasing fluid viscosity with Ficoll, we did not observe any rotating upstream flagella (Fig. 4D). Interestingly, when we reversed the direction of flow, we observed that some flagella began to rotate again (Fig. S11), indicating the block of rotation is reversible. Thus, the shear force associated with flow reversibly blocks rotation by bending upstream flagella around the cell body.

Here, we made two important discoveries: flagellar rotation promotes surface departure and shear force blocks rotation of upstream flagella. Combining these two ideas, we hypothesized that cells with upstream flagella would be less likely to depart the surface in flow. In support of our hypothesis, WT cells with upstream flagella were less likely (20%) to depart the surface than cells with downstream flagella (45%) (Fig. 4E). As type IV pili also promote surface departure, we repeated this experiment with Δ*pilA* cells. Similarly, Δ*pilA* cells with upstream flagella were less likely (1%) to depart the surface than cells with downstream flagella (19%) (Fig. 4F). Based on these data, we conclude that cells with their flagella facing upstream are less likely to depart the surface than cells with their flagella facing downstream. Together, our findings establish that shear force bends flagella, blocks rotation, and ultimately controls surface behavior of *P. aeruginosa*.

## DISCUSSION

**Fig 5:**
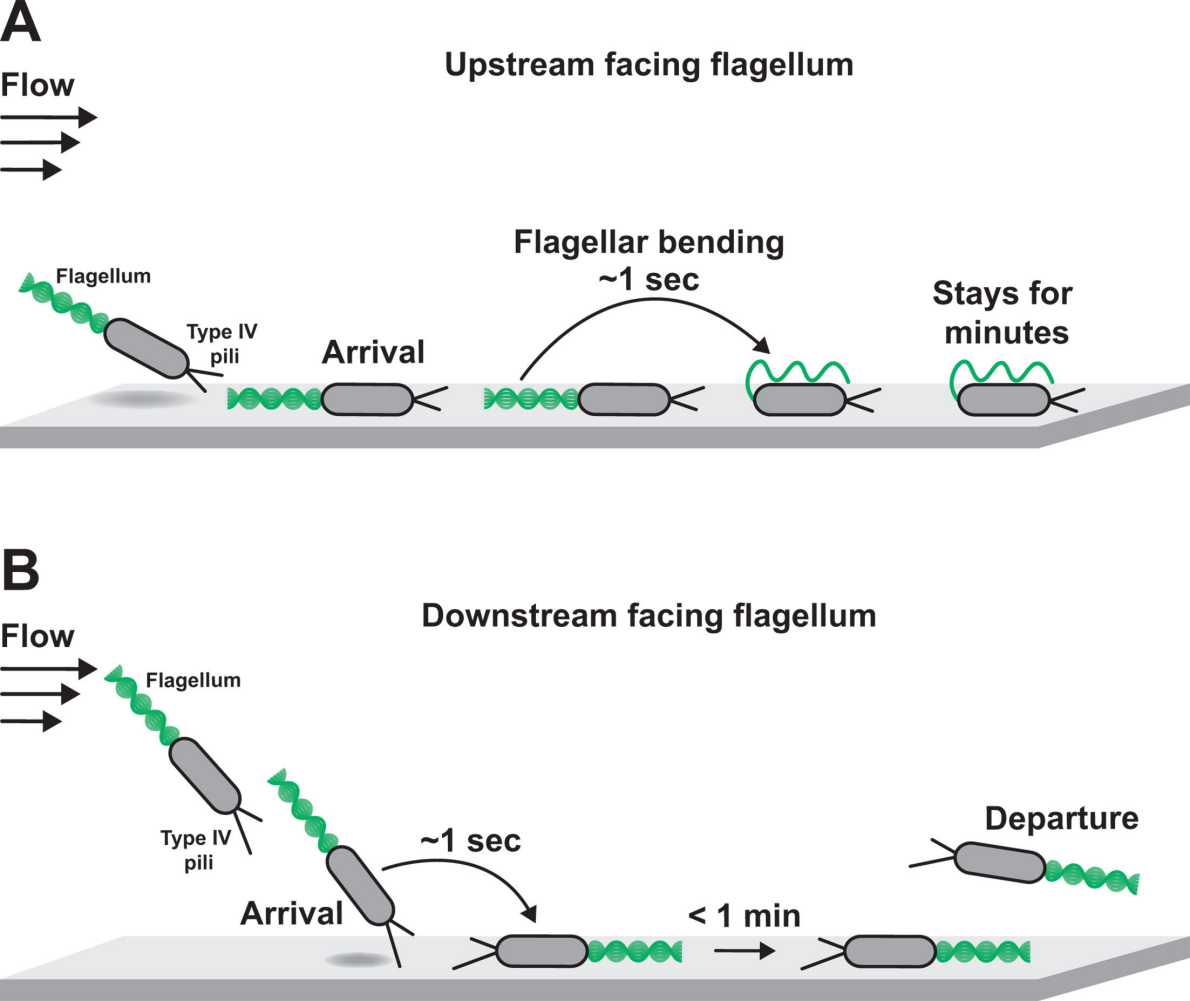
Flow-induced bending of flagella controls bacterial surface behavior. *P. aeruginosa* cells that arrive at the surface in flow can either have upstream facing (**A**) or downstream facing (**B**) flagella. (**A**) Cells can arrive on the surface with their flagella facing upstream into the flow. Quickly after arrival, the shear force associated with flow bends their flagella around their cell body and blocks rotation. As a consequence, these cells typically remain adhered to the surface. (**B**) Alternatively, cells can tip over as they arrive on the surface and have their flagella facing downstream. The flagella of these cells can continue to rotate, and these cells are likely to quickly depart the surface.

Our experiments demonstrate how flow and flagellar rotation interact to control surface departure of *P. aeruginosa*. While other studies have explored the impact of flagellar rotation on surface interactions without flow (16, 43, 44), our work highlights how flow and flagellar rotation interact in important ways. Using mutants with paralyzed flagella, we showed that flagellar rotation drives surface arrival in flow (Fig. 1). While tracking cells after surface arrival, we learned that flagellar rotation also promotes surface departure in flow (Fig. 2). By combining microfluidics and flagellar labeling, we discovered that flagella can rotate after surface arrival and cells with rotating flagella are more likely to depart the surface (Fig. 3). Additionally, we discovered that shear force bends upstream facing flagella and blocks their rotation (Fig. 4). Together, these experiments lead us to a model where cells with downstream facing flagella are more likely to depart the surface than cells with upstream facing flagella (Fig. 5). Our results highlight how environmental and cell-generated mechanics can interact to yield unexpected surface behaviors.

How does shear force block flagellar rotation? By independently changing flow intensity and solution viscosity, we discovered that increasing shear force blocks rotation of upstream facing flagella (Fig. 4D). Additionally, we discovered that increasing shear force bends upstream facing flagella (Fig. S8B). These results lead us to the hypothesis that flagellar bending traps the flagellar filament next to the cell, which physically prevents rotation. An alternative model where the cell “jump ropes” over a rotating flagellum is possible (45, 46), but we did not observe this behavior in our experiments. As we did not observe motion of blocked upstream flagella after a few seconds on the surface, we conclude that rotation had truly stopped. Thus, our data support a model where shear force bends upstream flagella, traps the filament between the surface and the cell, and ultimately blocks rotation.

How does flagellar bending block rotation? We have several hypotheses regarding the mechanism underlying rotational blockage. First, the load on the motor may be greater than the torque the motor can generate, which would lead to stalling (47–50). Second, a molecular brake or clutch may inhibit rotation by binding to the motor (38, 51–53). Third, mechanical bending of the filament could lead to transient motor disengagement by physically twisting the motor. Our current evidence is consistent with any of these hypotheses or potentially a combination of them. For example, bending of the flagella may lead to load-dependent stalling at early timescales and regulatory inhibition at late timescales. When load increases, the motor can recruit more stators and increase torque (7, 50, 54–56), but if the filament is physically trapped the motor may disengage. Additionally, cells may exhibit differences in load-dependent responses, which could lead to heterogeneity in downstream surface responses mediated by c-di-GMP levels (43, 50, 55, 57, 58).

How do environmental mechanics impact bacterial behavior? The data presented here support the conclusion that environmental shear force can directly impact behavior by bending cellular appendages. In our model, physical bending blocks flagellar rotation, which controls whether or not a cell departs the surface. In contrast, there is evidence that other forms of mechanoregulation require intracellular signaling. For example, mechanosensing of surface contact by type IV pili and flagella controls intracellular signaling that triggers synthesis of a surface adhesin (17, 18). Additionally, flow-driven transport of hydrogen peroxide overcomes cell scavenging to trigger an intracellular signaling pathway (21, 23). When comparing and contrasting flagellar bending, mechanosensing, and flow-driven transport, it becomes clear that environmental mechanics can impact bacterial behavior in ways that appear phenotypically similar but mechanistically different. Moving forward, researchers should continue to search for new forms of bacterial mechanoregulation while understanding that the mechanisms underlying these processes may be diverse and unique.

## MATERIALS AND METHODS

### Bacterial strains, plasmids, and growth conditions

Bacterial strains used in this study are described in Table S1, the primers used are described in Table S2, and the plasmids used are described in Table S3. *P. aeruginosa* strains were grown on LB agar plates (1.5% Bacto Agar) and in liquid LB in a roller drum at 37°C. LB medium was prepared using premix Miller LB Broth (BD Biosciences) and using standard LB preparation protocols.

### Generation of *P. aeruginosa* mutants

Gene deletions were generated using the lambda Red recombinase system as previously described (26). The deletion construct was Gibson-assembled from three PCR products. First fragment, approximately 500 bp upstream of the target insertion site was amplified from PA14 genomic DNA. Second fragment, containing *aacC1* ORF flanked by FRT sites was amplified from pAS03D. Third fragment, approximately 500 bp downstream of the target insertion site was amplified from PA14 genomic DNA. The Gibson-assembled product was transformed into PA14 cells expressing the plasmid pUCP18-RedS. The colonies were selected on 30 µg/mL gentamicin, the mutants of interest were counter-selected on 5% sucrose, and pFLP2 was used to flip out the antibiotic resistance gene. pUCP18-RedS and pFLP2 were selected for using 300 µg/mL carbenicillin.

### Construction of microfluidic devices

Microfluidic devices were created using soft lithography techniques as previously described (26). Devices were designed on Illustrator (Adobe Creative Suite), and masks were printed by CAD/Art Services. Molds were made on 100 mm silicon wafers (University Wafer) and spin coated with SU-8 3050 photoresist (MicroChem). Polydimethylsiloxane (PDMS) chips were plasma-treated to bond with glass slides for at least 24 h prior to experiments. The devices used in all experiments contained seven parallel channels (500 µm width × 50 µm height × 2 cm length). Channels individually contained an inlet tube and an outlet tube. PDMS chips were plasma treated to a 60 mm × 35 mm × 0.16 mm superslip micro cover glass (Ted Pella, Inc.).

### Residence time assays

Surface residence time experiments were performed as previously described (26). *P. aeruginosa* cells were loaded into plastic 5 mL syringes (BD) at mid-log phase with an optical density of approximately 0.5. All microfluidic experiments were performed at ~22°C. The device set-up involves the loaded syringes attached to tubing which connects the needle to the inlet of the device (BD Intramedic Polyethylene Tubing; 0.38 mm inside diameter, 1.09 mm outside diameter). These syringes were situated on a syringe pump (KD Scientific Legato 210) which was used to produce fluid flow. The outlet of the device employed the same tubing and vacated into a bleach-containing waste container. The syringe pump was used to generate flow rates of 10 µL/min, which correspond to shear rates of 800 s^−1^.

### Phase contrast microscopy

Images were obtained with a Nikon Ti2-E microscope controlled by NIS Elements as previously described (26). All images were taken with a Nikon 100× Plan Apo Ph3 1.45 NA objective, a Hamamatsu Orca-105 Flash4.0LT + camera, and Lumencor Sola Light Engine LED light source.

### Imaging of flagella

Strains were grown at 37°C overnight, back diluted 1:100, grown to early log phase, and then incubated with Alexa488-mal (VWR) for 45 min. Cells were washed twice using centrifugation to remove excess dye. *P. aeruginosa* cells were loaded into plastic 5 mL syringes (BD) at mid-log phase with an optical density of approximately 0.5. All microfluidic experiments were performed at ~22°C. The device set-up involves the loaded syringes attached to tubing, connecting the needle to the inlet of the device (BD Intramedic Polyethylene Tubing; 0.38 mm inside diameter, 1.09 mm outside diameter). Syringes were situated on a syringe pump (KD Scientific Legato 210) which was used to produce fluid flow. The outlet of the device employed the same tubing and vacated into a bleach-containing waste container. The syringe pump was used to generate flow rates of 10 µL/min, which correspond to shear rates of 800 s^−1^. For most fluorescence experiments, there was no starting, stopping, or pulsatility of fluid flow unless specified. In the experiment where fluid flow direction was reversed, the syringe pump’s pusher block was released. The syringe plunger was then manually pulled back to reverse the fluid flow direction in the microfluidic channel. To view flagella, we employed a Nikon 100×/1.45 objective on an inverted fluorescent Nikon Eclipse Ti2 microscope with an ORCA-fusion BT sCMOS camera, for image acquisition. Exposure time of 100 ms was used for fluorescence imaging of rotating flagella. Flagellar images were taken using Nikon Elements Software and analyzed using ImageJ.

### Swim assay

Strains were grown at 37°C overnight, and then 1 mL aliquots were transferred to 1.5 mL microcentrifuge tubes. Sterile toothpicks were used to stab into LB plates containing 0.3% agar, and plates were incubated overnight at 37°C. The diameter of the swim zones was measured using Adobe Illustrator.

### Shear rate and shear force calculations

The shear rate experienced in the microfluidic devices was calculated using the following equation:


$$Wall\;shear\;rate\;∼\frac{6Q}{{wh}^{2}}$$

Where *Q* is the flow rate, *w* is the channel width, and *h* is the channel height. Shear stress was calculated as the product of shear rate and viscosity. Shear force was calculated as the product of shear stress and the surface area of a cell, which was estimated to be 2.5 µm^2^ (9).

### Shear force modification assay

To adjust the viscosity of *P. aeruginosa* cell cultures, we prepared a solution containing a 15% concentration of the viscous agent Ficoll. All microfluidic experiments were performed at ~22°C. The device set-up involves the loaded syringes attached to tubing which connects the needle to the inlet of the microfluidic device (BD Intramedic Polyethylene Tubing; 0.38 mm inside diameter, 1.09 mm outside diameter). The loaded syringes were situated on a syringe pump (KD Scientific Legato 210) which was used to produce fluid flow. The outlet of the device employed the same tubing and vacated into a bleach-containing waste container. The syringe pump was used to generate flow rates of 1 µL/min (shear rate of 80 s^−1^) and 10 µL/min (shear rate of 800 s^−1^).

## SUPPLEMENTAL MATERIAL

Supplemental Material mbio.02740-25-s0001.docx Supplemental figures and tables.

ASM does not own the copyrights to Supplemental Material that may be linked to, or accessed through, an article. The authors have granted ASM a non-exclusive, world-wide license to publish the Supplemental Material files. Please contact the corresponding author directly for reuse.
