# Supplementary material for: Flow-induced bending of flagella restricts Pseudomonas aeruginosa surface departure
Source: mBio. 2025 Dec 12;17(1):e02740-25. doi: 10.1128/mbio.02740-25 (PMC12802323; doi:10.1128/mbio.02740-25)
Supplement: Supplemental Material — Supplemental figures and tables. [file mbio.02740-25-s0001.docx]

Supplementary Information for

**Flow-induced bending of flagella restricts *Pseudomonas aeruginosa* surface departure**

Jessica-Jae S. Palalay^1^, Joseph E. Sanfilippo^1^*

^1^Department of Biochemistry, University of Illinois at Urbana-Champaign, Urbana, IL, USA, 61801

*To whom correspondence should be addressed. Email: josephes@illinois.edu

**This includes:**

Supplemental Figures S1 to S11

Supplemental Tables S1 to S3

Supplemental References


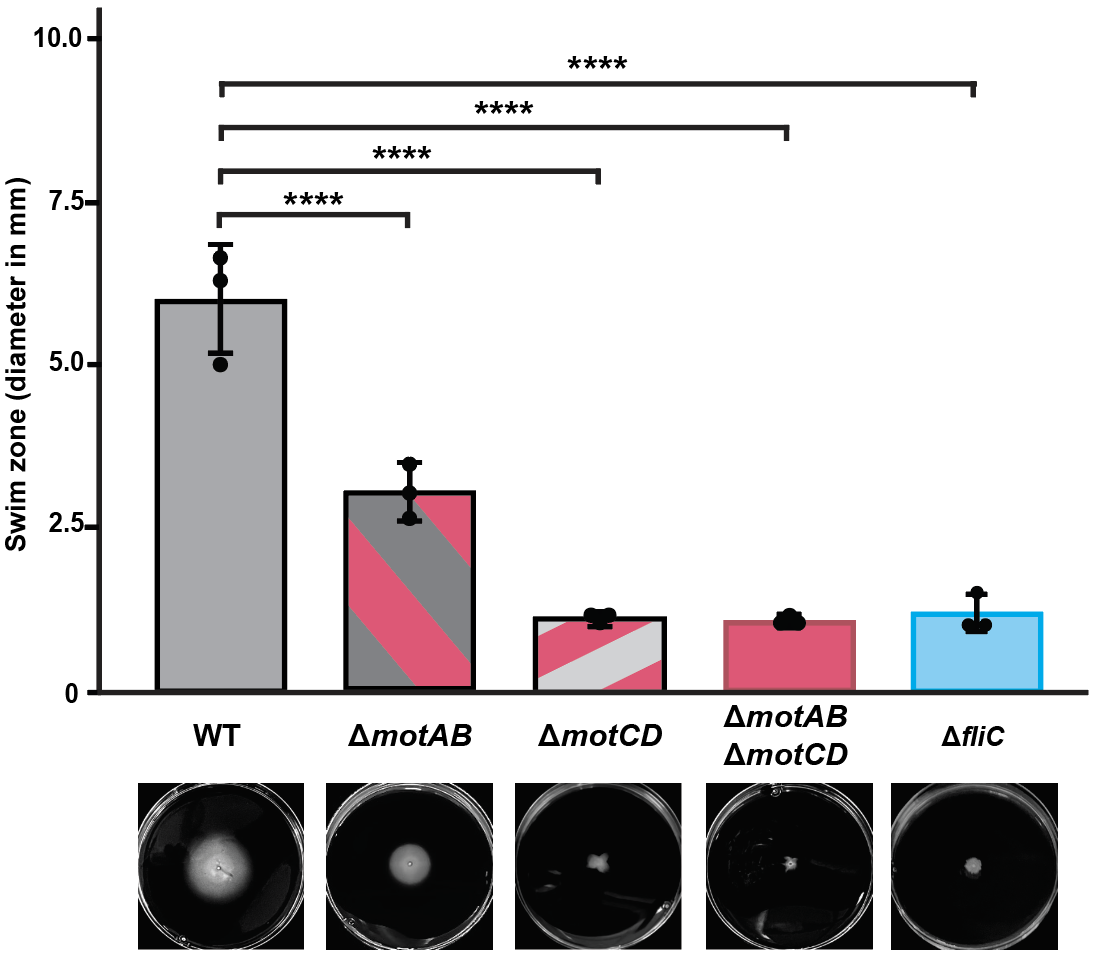


**Figure S1: MotAB and MotCD both contribute to swimming in agar motility assays.**

Quantification of swim zones for WT and flagellar mutant strains. The diameter of each resulting swim zones was measured after 18 hours at 37°C. Quantification shows the average and SD of three biological replicates. Statistical significance determined using one-way ANOVA followed by Dunnett’s test; ****, *P* < 0.0001. Representative swim plate images for each strain were captured after 18 hours.


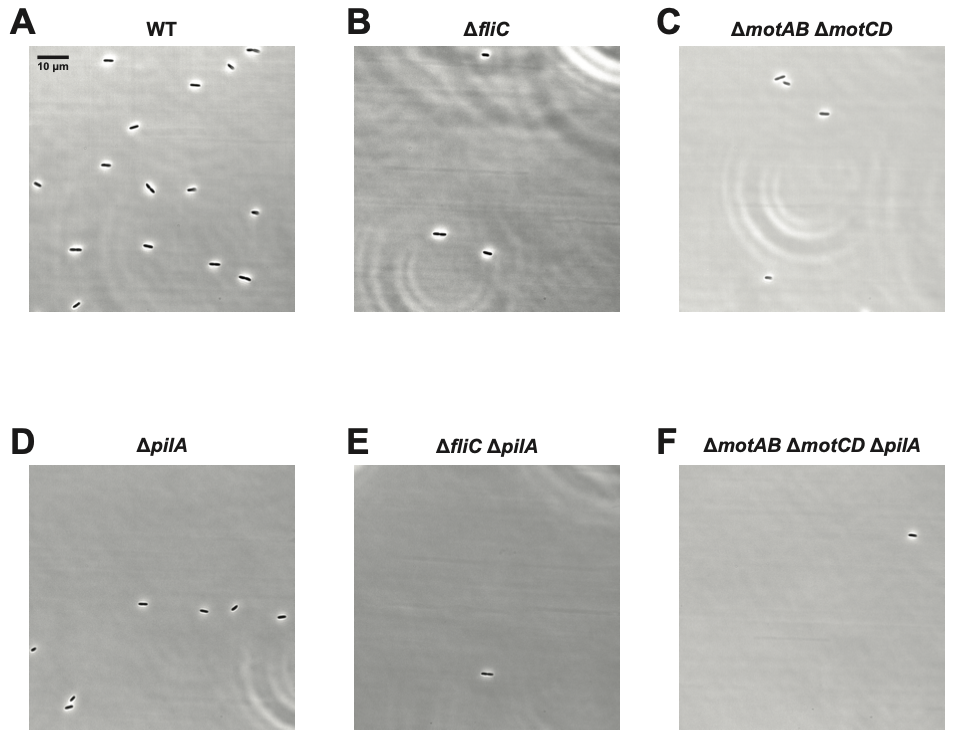


**Figure S2: Flagellar rotation promotes *P. aeruginosa* surface arrival in flow.**

Representative phase images showing cells after they have arrived on the glass surface of the microfluidic device. Cells were flowed into the channel at a shear rate of 800 s^-1^. Images were taken after 30 seconds of flow. Scale bar, 10 μm. Quantification of these experiments is shown in Figure 1B.


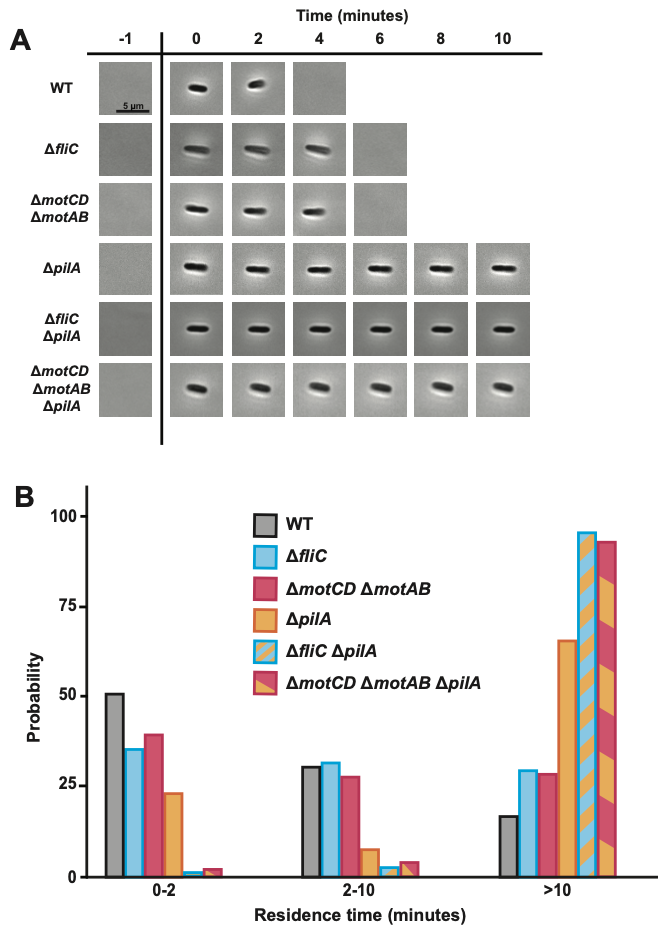


**Figure S3: Flagellar rotation promotes *P. aeruginosa* surface departure in flow.**

(A) Representative phase images of WT and mutant cells that arrived on the surface after being flowed into microfluidic devices at a shear rate of 800 s^-1^. Scale bar, 5 μm. (B) Probability of the WT and mutant cell surface residence times from Figure 2 categorized into three groups (0-2 minutes, 2-10 minutes, and >10 minutes). Three biological replicates were performed and 150 cells (50 from each replicate) of each bacterial strain were chosen at random for quantification.


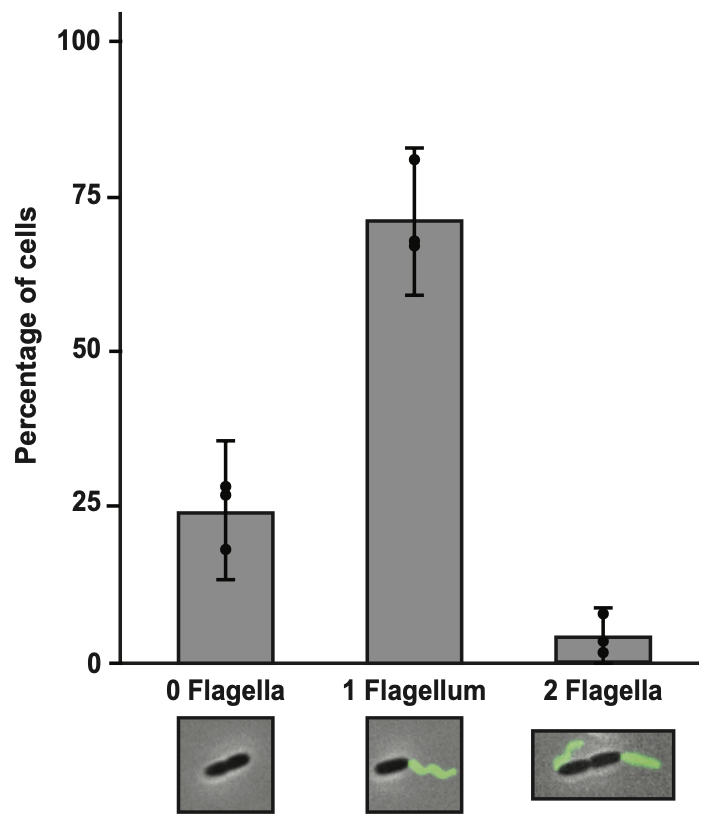


**Figure S4: WT *P. aeruginosa* cells typically have 1 flagellum.**

Quantification and representative images of surface-attached WT cells of three categories (no labeled flagella, one labeled flagellum, or 2 labeled flagella). Quantification shows the average and standard deviation of three biological replicates.


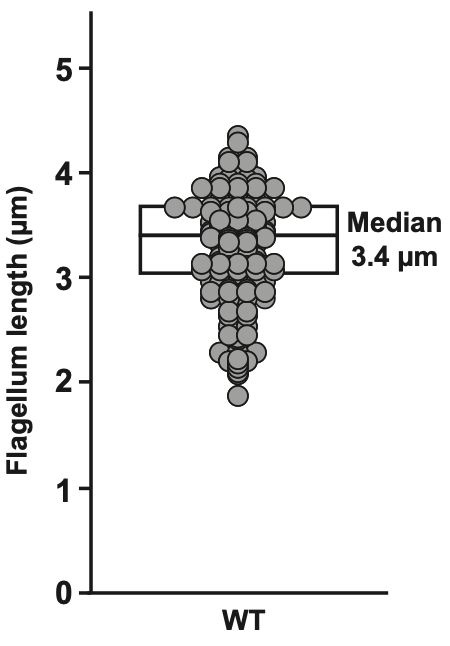


**Figure S5: Flagella of WT *P. aeruginosa* cells are typically 3.4 µm long.**

Quantification of flagellar length of fluorescently labeled WT cells. Flagellar length was measured from base to tip. Each data point represents the length of one flagellum. Three biological replicates were performed and 200 cells were chosen at random for quantification. The boxplot represents the 25^th^ percentile, median, and 75^th^ percentile.


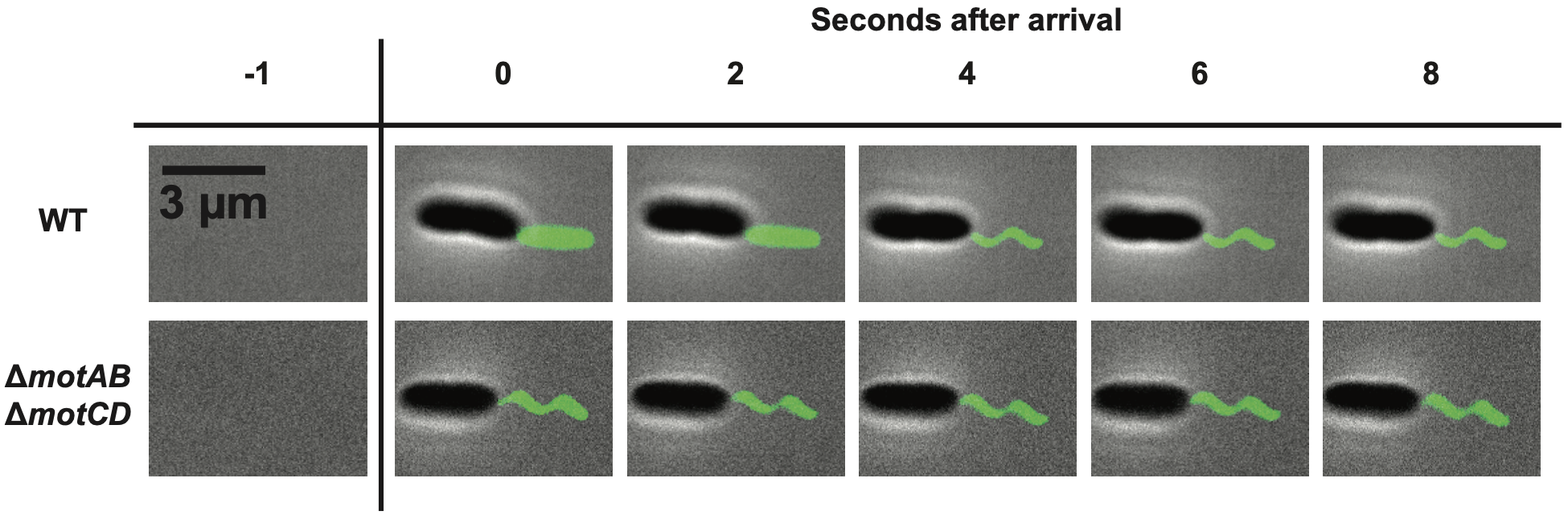


**Figure S6: Zoomed in images of fluorescently labeled flagella.**

Representative images of surface-attached WT (top) and ∆*motAB* ∆*motCD* (bottom) cells with fluorescently labeled flagella. WT cell has a rotating flagellum, having a green rectangular-shaped blur positioned next to the cell. ∆*motAB* ∆*motCD* cell does not have a rotating flagellum, having a green waveform shape positioned next to the cell. Scale bar, 3 µm.


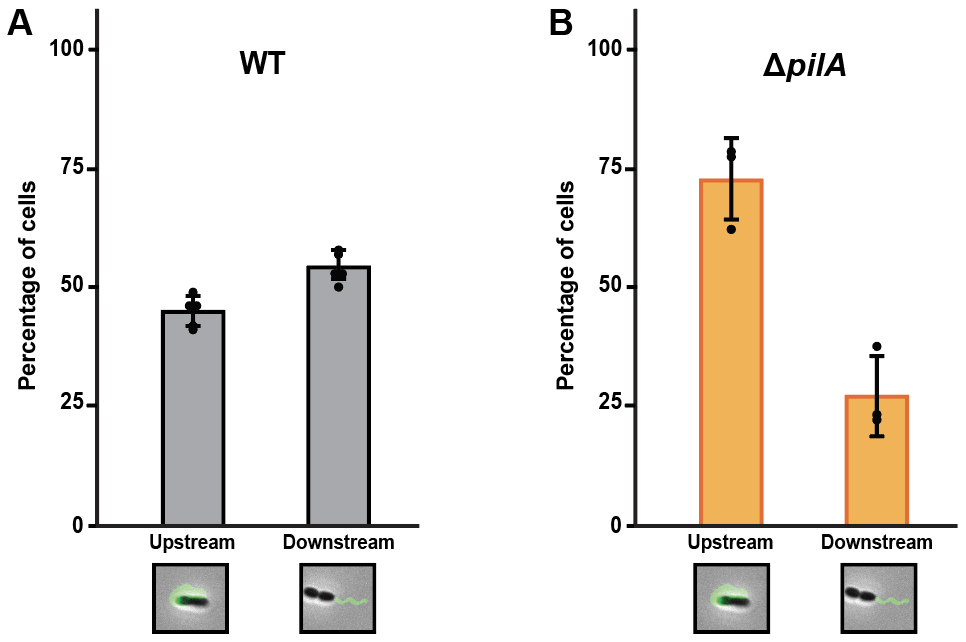


**Figure S7: Cells having type IV pili are equally likely to arrive with upstream or downstream facing flagella.**

Quantification and representative images of surface-attached WT cells (A) and surface-attached pilus mutant (∆*pilA*) cells (B) with upstream facing flagella or downstream facing flagella. Quantification shows the average and standard deviation of five biological replicates (WT) and three biological replicates (*ΔpilA*).


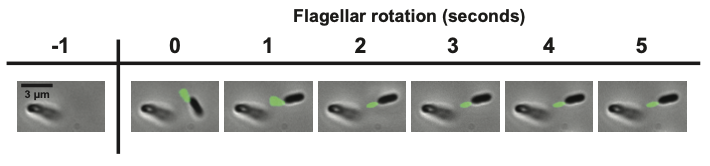


**Figure S8: Cell-cell shielding can prevent flow-induced flagellar bending.**

Images of a surface-attached WT cell with an upstream facing flagellum. Cell was flowed in at a shear rate of 800 s^-1^. Cell arrives on the surface at 0 seconds and continues to rotate its flagellum for at least the next 5 seconds. Scale bar, 3 µm.


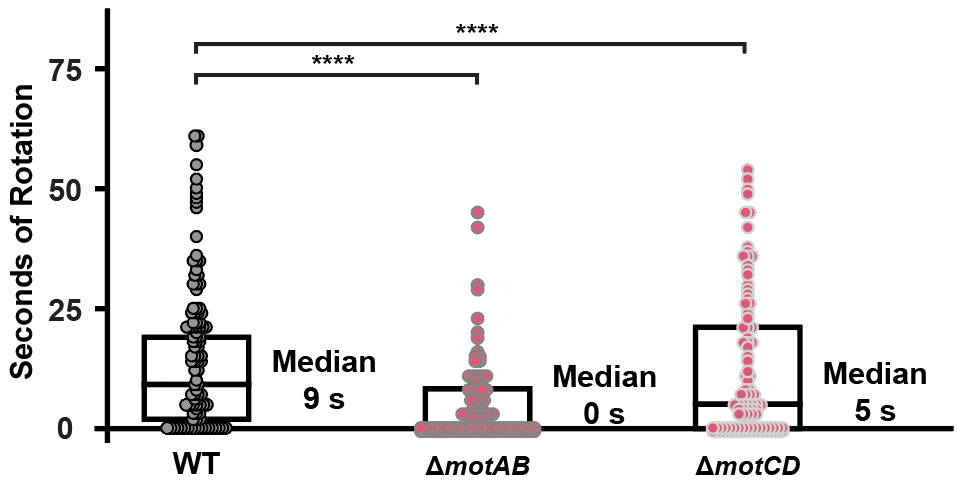


**Figure S9: MotAB and MotCD both contribute to flagellar rotation on surfaces in flow.**

Duration of flagellar rotation of WT, *ΔmotAB*, and *ΔmotCD* cells having downstream facing flagella. Each data point represents one flagellum. Three biological replicates were performed and 100 WT, 100 *ΔmotAB* and 100 *ΔmotCD* cells were chosen for quantification. Boxplot represents the 25^th^ percentile, median, and 75^th^ percentile. Statistical significance determined using one-way ANOVA followed by Dunnett’s test; ****, *P* < 0.0001.


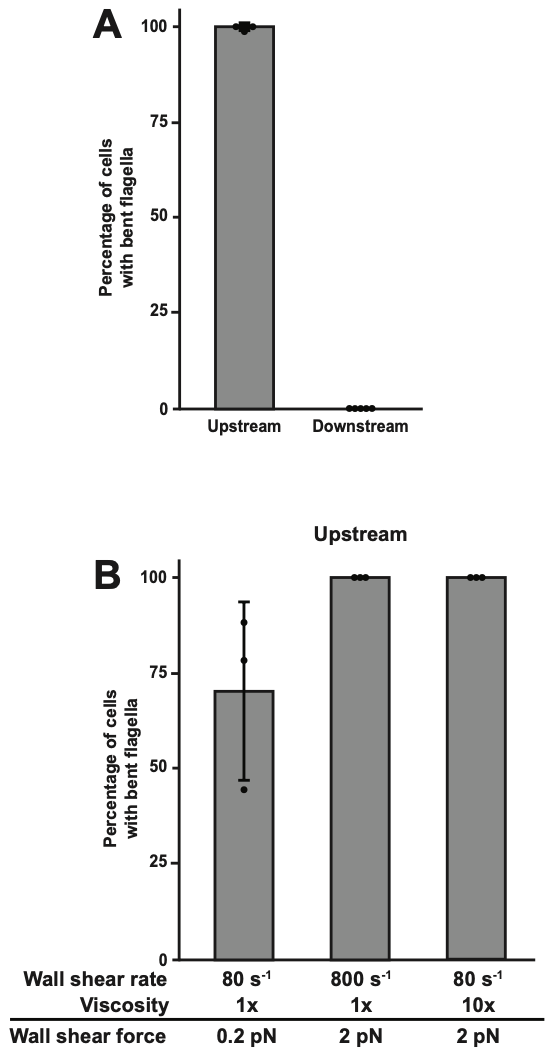


**Figure S10: Shear force bends upstream facing flagella around the cell.**

(A) Percentage of WT cells with bent flagella. Cells were flowed in at a shear rate of 800 s^-1^ and categorized as having upstream facing or downstream facing flagellum. Quantification shows the average and SD of five biological replicates. (B) Percentage of upstream facing flagella that bent around cells during exposure to different shear forces. Shear force was increased either by changing shear rate or fluid viscosity. Shear rate was modified by changing the flow rate of the syringe pump. 10x viscosity was generated by adding 15% Ficoll, which has been shown previously to modify local viscosity (9, 42). Quantification shows the average and SD of three biological replicates.


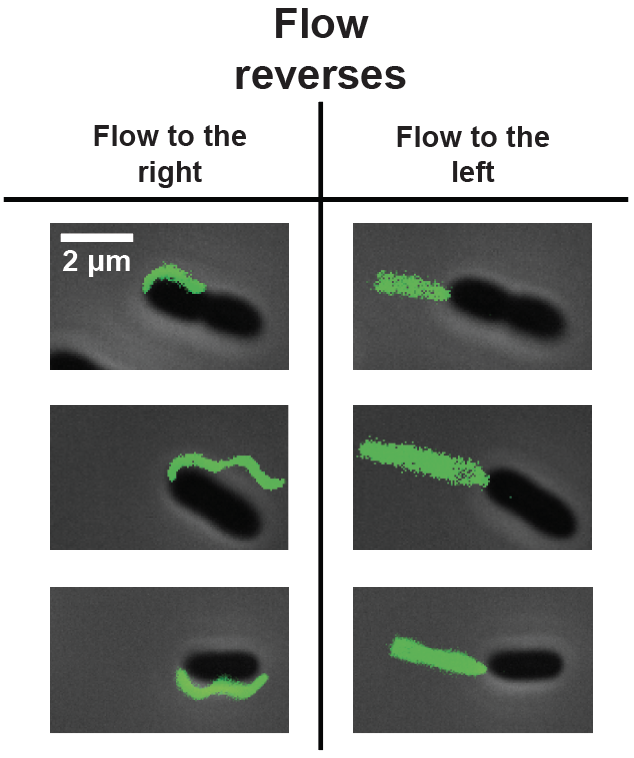


**Figure S11: Reversing fluid flow can unbend upstream facing flagella and allow the cell to resume flagellar rotation.**

Images are representative examples of surface-attached WT cells with fluorescently labeled flagella. Under conditions with flow moving from left to right, upstream facing flagella bend around the cell. When fluid flow stops, flagella can unbend and straighten out. Flagellar rotation can resume as fluid flow is reversed. This figure shows three WT cells (top, middle, and bottom) before and after flow reverses. Scale bar, 2 µm.

**Table S1: *P. aeruginosa* strains used in this study**

| ***P. aeruginosa* strain** | **Description** | **Source** |
| --- | --- | --- |
| PA14 | wildtype; clinical isolate from burn wound | (59) |
| JS177 | Δ*pilA::aacC1* | (26) |
| JS176 | Δ*pilA::FRT* | (26) |
| JS191 | Δ*fliC::aacC1* | This paper |
| JS190 | Δ*fliC::FRT* | This paper |
| JS197 | Δ*motCD::aacC1* | This paper |
| JS200 | Δ*motCD::FRT* | This paper |
| JS175 | Δ*motAB::aacC1* | This paper |
| JS174 | Δ*motAB::FRT* | This paper |
| JS206 | Δ*fliC::FRT* Δ*pilA::aacC1* | This paper |
| JS212 | Δ*fliC::FRT* Δ*pilA::FRT* | This paper |
| JS205 | Δ*motCD::FRT* Δ*motAB::aacC1* | This paper |
| JS213 | Δ*motCD::FRT* Δ*motAB::FRT* | This paper |
| JS318 | Δ*motCD::FRT* Δ*motAB::FRT* Δ*pilA::aacC1* | This paper |
| JS319 | Δ*motCD::FRT* Δ*motAB::FRT* Δ*pilA::FRT* | This paper |
| JS115 | *fliC* T394C; Cysteine knock-in mutant for flagellar labeling | This paper |
| JS327 | *fliC* T394C Δ*motCD::FRT* Δ*motAB::aacC1* | This paper |
| JS328 | *fliC* T394C Δ*motCD::FRT* Δ*motAB::FRT* | This paper |
| JS336 | *fliC* T394C Δ*motAB::aacC1* | This paper |
| JS335 | *fliC* T394C Δ*motAB::FRT* | This paper |
| JS325 | *fliC* T394C Δ*motCD::aacC1* | This paper |
| JS326 | *fliC* T394C Δ*motCD::FRT* | This paper |
| JS334 | *fliC* T394C Δ*pilA::FRT* | This paper |

**Table S2: Plasmids used in this study**

| **Plasmid** | **Description** | **Source** |
| --- | --- | --- |
| pAS03D | Plasmid to generate deletion mutants in *P. aeruginosa* PA14 | (60) |
| pFLP2 | Plasmid expressing FLP2 to recombine FRT sites | (61) |
| pUCP18-RedS | Lambda red recombineering vector | (60) |

**Table S3: Primers used in this study**

| **Primer** | **Sequence (5'-3')** |
| --- | --- |
| *ΔpilA*-1 | cgcagtaggcgataccgaat |
| *ΔpilA*-6 | aggaactcggttttctccgc |
| *ΔfliC*-1 | ctgctatcgcgacagtctcc |
| *ΔfliC*-6 | aatcggtcgagcctactcct |
| *ΔmotCD*-1 | ggtgctgatccagcacatgcc |
| *ΔmotCD*-2 | ccatcgaaggcagtctcctcattcc |
| *ΔmotCD*-3 | **gcaggaatgaggagactgccttcgatggccacccacgtga**attccggggatccgtcgacc |
| *ΔmotCD*-4 | **tggccagttcggccccggcgcccgtacgcaaaccatgttt**gtgtaggctggagctgcttc |
| *ΔmotCD*-5 | gcgcgaaacatggtttgcgtac |
| *ΔmotCD*-6 | cattgaccatcagcacgccgag |
| *ΔmotAB*-1 | cagtggatttcctgccagagc |
| *ΔmotAB*-2 | gaggaccggacgtgcgaaatg |
| *ΔmotAB*-3 | **tccccctccttatcctgttcatttcgcacgtccggtcctc**attccggggatccgtcgacc |
| *ΔmotAB*-4 | **gcacattctggcaagcatgccggaaacggactactgaacc**gtgtaggctggagctgcttc |
| *ΔmotAB*-5 | ggttcagtagtccgtttccggc |
| *ΔmotAB*-6 | catcgatgcgctgctgaatgt |

**Supplemental References**

59. L. G. Rahme, *et al.*, Common virulence factors for bacterial pathogenicity in plants and animals. *Science* **268**, 1899–1902 (1995).

60. A. Siryaporn, S. L. Kuchma, G. A. O’Toole, Z. Gitai, Surface attachment induces *Pseudomonas aeruginosa* virulence. *Proc. Natl. Acad. Sci.* **111**, 16860–16865 (2014).

61. T. T. Hoang, R. R. Karkhoff-Schweizer, A. J. Kutchma, H. P. Schweizer, A broad-host-range Flp-FRT recombination system for site-specific excision of chromosomally-located DNA sequences: application for isolation of unmarked *Pseudomonas aeruginosa* mutants. *Gene* **212**, 77–86 (1998).
